# Optimizing the Energy and Throughput of a Water-Quality Monitoring System

**DOI:** 10.3390/s18041198

**Published:** 2018-04-13

**Authors:** Segun O. Olatinwo, Trudi-H. Joubert

**Affiliations:** Department of Electrical, Electronic and Computer Engineering, University of Pretoria, Pretoria 0001, South Africa; trudi.joubert@up.ac.za

**Keywords:** energy harvesting, energy efficiency, water-quality monitoring, wireless sensor network, optimization

## Abstract

This work presents a new approach to the maximization of energy and throughput in a wireless sensor network (WSN), with the intention of applying the approach to water-quality monitoring. Water-quality monitoring using WSN technology has become an interesting research area. Energy scarcity is a critical issue that plagues the widespread deployment of WSN systems. Different power supplies, harvesting energy from sustainable sources, have been explored. However, when energy-efficient models are not put in place, energy harvesting based WSN systems may experience an unstable energy supply, resulting in an interruption in communication, and low system throughput. To alleviate these problems, this paper presents the joint maximization of the energy harvested by sensor nodes and their information-transmission rate using a sum-throughput technique. A wireless information and power transfer (WIPT) method is considered by harvesting energy from dedicated radio frequency sources. Due to the doubly near–far condition that confronts WIPT systems, a new WIPT system is proposed to improve the fairness of resource utilization in the network. Numerical simulation results are presented to validate the mathematical formulations for the optimization problem, which maximize the energy harvested and the overall throughput rate. Defining the performance metrics of achievable throughput and fairness in resource sharing, the proposed WIPT system outperforms an existing state-of-the-art WIPT system, with the comparison based on numerical simulations of both systems. The improved energy efficiency of the proposed WIPT system contributes to addressing the problem of energy scarcity.

## 1. Introduction

Clean water is indispensable for human survival as it is used for drinking and hygiene and in agriculture and industry. The increase in anthropogenic activities in recent years has had a tremendously negative impact on the environment, and this has contributed to a low quality of water in terms of physical, microbiological and chemical properties. Consequently, the constant monitoring of water quality has become a necessity for safety of lives as stipulated by various standards organizational bodies such as the World Health Organization (WHO) and the European Union (EU). Monitoring guards against the supply of contaminated water. The traditional method used for water monitoring is confronted by several problems ranging from high cost, requirement of off-site analysis, time wasting, to interference from operators. To address these problems in a timely manner, wireless sensor network (WSN) technology is a promising economical approach which involves the deployment of different sensor nodes measuring water quality at a desired water-processing station. Monitoring parameters might typically include inorganic and organic contaminants [1,2,3] such as pH detection, dissolved metal ion detection, and bacterial load detection. The data collected by the water-quality sensor nodes regarding the quality of the water at the water-processing station is transmitted to various data centers in a timely manner through internet technology. The data centers analyses the water-quality data. Based on the analysis, necessary decisions are made. For example, in a case of contamination, an emergency alarm is given, and such water is prevented from being distributed. WSNs are not only valuable tools employed in water-quality monitoring [4], but also in various applications such as water leakage monitoring [5] and traffic monitoring [6], to collect, process and disseminate environmental data to various data centers [7].

Energy scarcity is a general problem in WSNs, and has been a major setback to the widespread deployment of various WSN applications over the years [8]. This is due to the limited energy capacity of sensor nodes because the energy storage devices in sensor nodes have a limited energy budget [7,8,9]. To address the problem of energy scarcity in WSNs and to make WSN applications sustainable, energy harvesting (EH) is a promising technique [10]. Consequently, WSN researchers have exploited and investigated various renewable energy sources that include radio frequency (RF) waves [11], wind [12] and solar [13] for energy harvesting. Among the identified renewable energy sources, a RF energy source is controllable [14], while other energy sources are uncontrollable [13]. This makes RF EH a promising technique. Furthermore, RF EH is advantageous compared to other methods because RF energy sources are reliable. Also, the form factor of a sensor node does not need to be increased while embedding the circuit of an RF energy harvester, providing a platform for portable sensor nodes. For these reasons, a system that transfers information by RF, but also harvests energy from RF waves, presents a promising method, known as wireless information and power transfer (WIPT). However, the existing EH WSN systems based on WIPT are plagued by the doubly near–far problem [15]. This paper therefore focuses on a new type of WIPT system that addresses this problem, specifically for use in energy-efficient water-quality monitoring WSN applications in an enclosed environment. 

RF energy sources are of two basic types, namely ambient RF energy sources (ARFES) and dedicated RF energy sources (DRFES) [14]. Examples of ambient RF energy sources are Wi-Fi routers, television transmitters, and radio transmitters [13], while examples of commercially available DRFES are cota^®^ transmitters and powercaster^®^ transmitters [16,17]. These dedicated RF power solutions are suitable for any location. Variants of Powercaster transmitters are RF power source TX91501 [18], and battery-powered wireless transmitters [18]. These transmitters can be used to reliably send RF energy wirelessly to sensor nodes up to 24 m away by using the unlicensed industrial, scientific and medical (ISM) frequency bands [19]. ARFES are unpredictable, while DRFES are predictable. Without efficient energy storage technology, the existing RF EH solutions based on ambient RF energy sources are not sustainable due to the unpredictable and the time-varying profile of ARFES [20]. To make the operation of WSN applications to be ever sustainable, RF EH from multiple DRFES is a promising technique [17]. However, the current RF EH solutions that employed multiple DRFES are confronted by EH unfairness as a result of the doubly near–far condition, resulting in a reduced system information-transmission rate (throughput) [15]. To seek a solution to these problems, the energy harvested and the information-transmission rate of each sensor node can be jointly optimized in the network [21], consequently also improving the problem of energy scarcity. Currently, there are four categories of RF receiver architectures employed in WIPT systems for RF EH and information transmission. The categories are the co-located RF receiver architecture, which includes power splitting and time-switching RF architectures, the separated RF receiver architecture, and the integrated RF receiver architecture [14]. Among the aforementioned RF receiver architectures, a time-switching RF receiver architecture is effective for RF EH, while also eliminating the need for unnecessary space, thus making a sensor node more compact [22]. However, the traditional RF-based EH systems using the time-switching RF receiver architecture are confronted by unfairness in EH among sensor nodes, unfairness in the allocation of information-transmission rates among sensor nodes, inefficient management of multiple DRFES among multiple sensor nodes, and susceptibility to wireless interference [23]. These problems are efficiently addressed in this paper by studying a multi-network, multi-sensor, and multi-source (MNMSMS) system for powering a water-quality monitoring system, while the goals of fair EH, interference mitigation, and fair allocation of information-transmission rates, among the sensor nodes are ensured. Furthermore, the essence of this study is to ensure that the sensor nodes are able to harvest a sufficient amount of energy required to transmit their sensed water-quality data to the data base station with the expected quality of service (QoS), while the achievable throughput of the system is also increased. The key contributions of this work include:

- The formulation of a time-division multiple access (TDMA) model for EH and information transmission for a multi-network, multi-sensor, and multi-source (MNMSMS) system.
- Based on the TDMA protocol proposed, we jointly optimize the EH time and the information-transmission time of each sensor node *n* and *m* in the downlink (DL) channels and the uplink (UL) channels respectively, by maximizing the sum-throughput of the MNMSMS system using convex optimization methods [24].
- Efficient multi-network, multi-sensor, DRFES selection, allocation, and information-transmission algorithms were developed for efficient DRFES selection and allocation for fair EH among sensor nodes *n* and *m*, and fair information-transmission rate allocation.

To the best of our knowledge, there is no work on a multi-network, multi-sensor, and multi-source RF EH system for water-quality monitoring WSNs.

This work is divided into the following sections: Section 2 gives an overview of water-quality monitoring system. Section 3 gives an insight of the water-quality sensor node architecture. Section 4 presents the related works on WIPT systems with a focus on the optimization of EH time and information-transmission rate. Section 5 presents the model of the proposed multi-network, multi-sensor, multi-source system in a static environment, and the TDMA model proposed. Section 6 presents the mathematical formulations to optimize the TDMA protocol proposed for the system. Section 7 presents the energy-harvesting and information-transmission rate joint optimization problem. Also, efficient algorithms for DRFES selection, allocation, and information transmission are discussed. Section 8 presents the system simulation results based on the achievable sum-throughput and fairness to validate the sum-throughput optimization problem. Section 9 concludes this work.

## 2. Overview of Water-Quality Monitoring System

In recent years, there has been an increase in the rate of urbanization and this has negatively affected the assurance of clean water in our society due to human activities and population growth [25,26]. Examples of activities that pollute water environments are toxic run-off from industrial waste, inorganic chemicals from factory processes, thermal pollution and mining [27]. These activities increase the acidity of water, resulting in a negative impact on the ecosystem. This has necessitated the continuous monitoring of water in urban areas in order to provide clean water to society. Therefore, this work focuses on water-quality monitoring in an urban area. Clean water is crucial for human health, good air, plant health, and animal health. Typically, water can be obtained from different water sources (e.g., groundwater, rivers, reservoirs), and processed by water plants using water-treatment techniques according to the guidelines of the international regulation bodies such as WHO for various purposes such as fish farming and drinking. After the water-treatment processes, water is further tested using water-quality sensors for any contaminants before it is distributed. Water-quality sensors are powerful and portable devices that can employ WSNs to remotely monitor the quality of water at different water-processing stations. Water-quality monitoring provides information on any changes in the water quality due to contamination or ageing of utilities, and prevents the distribution of polluted water to consumers.

The water-quality sensor market has experienced tremendous growth in recent years. In 2015, it was valued at $2.95 billion USD, and the market size has been envisioned to grow up to $4.69 billion USD by 2025, at an annual rate of 4.54% [28]. The growth in the water-quality sensor market size is due to the wide adoption of WSN technology for water-quality monitoring across the world, and several technological improvements in the performance of water-quality sensors. The improvement in low-power electronics has given impetus to water-quality sensors that require supply power in the range of micro-Watt and milli-Watt to perform the measurement, processing and transmission of water-quality data. For instance, the MSP430F1611 microcontroller manufactured by Texas Instruments (TI) used in water-quality applications consumes 330 μA power in the active state, 1.1 μA in the standby state, and 0.2 μA in the idle state [29]. 

## 3. Water-Quality Sensor Node Architecture

The proposed WSN system devoted to the monitoring of water quality at a water-processing station is composed of water-quality sensor nodes which contain a low-power sensor module, an ultra-low power micro controller (MCU) module such as TI MSP430F1611 (Dallas, TX, USA), and a low-power RF transceiver module such as TI CC2420 (Dallas, TX, USA) [30]. The sensor nodes are wirelessly powered by dedicated RF sources such as the Powercaster transmitter TX91501 (Pittsburgh, PA, USA) [19,22]. The sensor module typically consumes minute amounts of energy, which is often negligible compared to the MCU and the RF transceiver. The dedicated RF sources have a fixed supply power [31]. The RF transceiver is a low-power compliant chip [30]. The water-quality sensor node and the RF power source exchange data using the IEEE 802.15.4 RF transceiver on an unlicensed ISM frequency band, such as 915 MHz. The MCU is a processing device that controls the entire operation of the sensor node. The MCU achieves the coordination of collection of the measured water-quality data from the sensor module, storing the collected data in the memory, and sending the stored data to the base station through the RF transceiver. An RF energy harvester module such as Powercast P2110 (Pittsburgh, PA, USA), and an energy storage module such as a Samxon supercapacitor is used to harvest energy in form of an RF signal from the RF power source and converts it into direct current (DC) power through an RF-to-DC rectifier [31]. The DC power is then stored in the energy-storage module to power the water-quality sensor node. To reduce the total energy consumption of the sensor nodes, the energy harvested and the information-transmission rate are jointly optimized to minimize the energy consumed by the sensor nodes, using the sum-throughput optimization method. For example, a fraction of the energy harvested by each sensor node is used to transmit its independent information to the base station in UL, while the remaining energy is used to run other modules.

## 4. Related Work

The concept of WIPT has recently gained particular attention in the research community due to continuous growth in the wireless recharging of WSNs. However, the existing solutions developed for WSN systems based on the WIPT concept are confronted by EH unfairness, and unfair allocation of the information-transmission rate among sensor nodes [23]. In an attempt to balance the trade-off between EH time and information-transmission rate allocation, a few solutions have been developed. For example, in [32], the authors developed a single-network, multi-sensor, single-source WIPT system. The authors considered concurrent EH and information transmission in the same frequency channel, and employed a cancellation scheme to reduce the effect of self-interference due to both these functions being in the same channel at the same time. In [33], a wireless body area network WIPT system was developed. The authors proposed two different protocols to optimize the information-transmission rate of each sensor node to the single access point through balancing the EH time and the information-transmission time of their WIPT system. In [34], the authors developed a single-network, multi-sensor, single-source WIPT system for implementation in a dynamic scenario. To address the problem of unfair information-transmission rate allocation among the sensor nodes, the authors employed a sum-throughput approach to maximize their WIPT system throughput based on fading channel power gain with a single DRFES. In [22], the authors developed a WIPT system with a single multi-sensor network, but with multiple sources. The problem of unfair information-transmission rate allocation among sensor nodes is addressed by maximizing the sum-throughput of their WIPT system, based on the fading channel power gains associated with multiple DRFES.

The problem of EH unfairness and unfair information-transmission rate allocation in WIPT systems may be improved beyond the solutions presented in [22,32,33,34]. The multi-sensor, multi-source WIPT system proposed in this paper expands on [22] by deploying multiple DRFES to address the EH unfairness problem between multiple groups of sensor nodes in a static environment, by employing a TDMA protocol and a sequential EH and transmit protocol. In [22], the sensor nodes deployed near to the DRFES harvest more energy compared to the sensor nodes deployed far from the DRFES due to the doubly near-far problem [15]. Consequently, there is EH unfairness between the sensor nodes in the single-network WIPT system. In the proposed WIPT system, the sensor nodes that ought to be far from the DRFES are deployed in another class of network than those close to the DRFES, based on a maximum distance threshold between the DRFES and the sensor nodes. 

## 5. Materials and Methods 

### 5.1. System Architecture 

**Figure 1:**
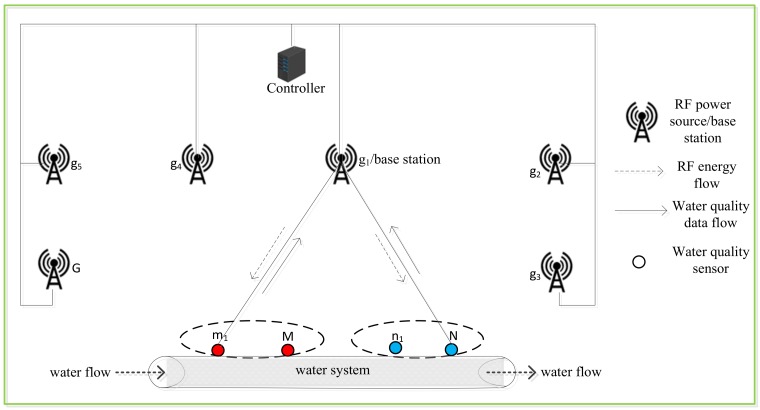
System model for a multi-network wireless information and power transfer (WIPT) system.

In the system model, we consider an EH WSN that consists of a multi-network, multi-sensor, and multi-source WIPT system. Let $\left\{n_{1},\;n_{2},\ldots ,\;N\right\}$ denote the set of water-quality sensor nodes *n* in Network 1, and $\left\{m_{1},\;m_{2},\ldots ,\;M\right\}$ denote the set of water-quality sensor nodes *m* in Network 2. The sensor nodes *n* and *m* are deployed in a predetermined manner at some strategic positions for effective monitoring and acquisition of vital parameters in a water-monitoring site as shown in Figure 1. The body of the water to monitor is channelled into an improvised man-made water-monitoring section in an enclosed environment that provides a suitable platform for the attachment of the water-quality sensors, and a continuous flow of water as in [35]. Let $\left\{g_{1},\;g_{2},\ldots ,\;G\right\}$ denotes the set of DRFES operating in a licence-free ISM band. Since the sensor nodes are deployed in a predetermined manner, their distance to the DRFES can be easily controlled. To achieve this, a multi-network WIPT system is considered. In the system, only $g_{1}$ represents both a DRFES and an information receiver or base station. This is capable of both energy transmission and information reception to and from sensor nodes n and m, while other DRFES g can transmit energy to the sensor nodes *n* and *m* in their allocated time based on the knowledge of channel-state information (CSI). To avoid the problem of destructive interference often experienced in the channels in multi-source systems due to multiple energy transmissions by the DRFES, a controller is employed. The controller connects all the DRFES to itself and controls when a DRFES g will transfer energy to the sensor nodes n and m, thus eliminating the possibility of the occurrence of any destructive interference. Also, the controller turns the DRFES g on and off at the calculated time-slots based on the channel conditions using the proposed DRFES selection algorithm which is implemented on the controller. Furthermore, the energy received by each sensor node *n* and m in each EH time-slot is reported by each sensor node to the controller. 

**Figure 2:**
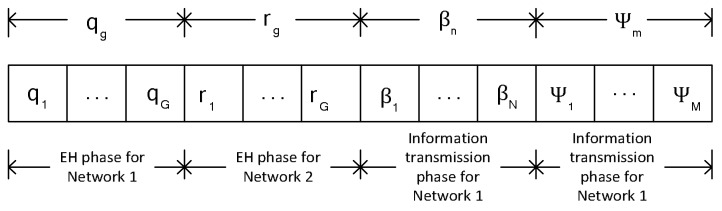
Proposed energy harvesting and information-transmission time-division multiple access (TDMA) model.

The sensor nodes *n* and *m* are connected in a single-hop manner, and each of the sensor node sends its independent information about the water-quality parameters of the site that include pH, bacteria, to the base station $g_{1}$. The base station $g_{1}$ delivers the received information to a water-quality monitoring center through a gateway connected to an Internet service. Each of the sensor nodes *n* and m in networks 1 and 2 is equipped with an omnidirectional antenna that operates on the TDMA model in Figure 2, such that either the information receiver or the RF energy harvester is connected to the antenna at a given time.

To prolong the network lifetime of the WIPT system, in each information-transmission time-slot, a fraction of the energy harvested is used for information transmission, while the remaining energy is stored in the energy storage, and it is used for running the basic operations of each sensor node. To avoid interference in EH and information transmission among the sensor nodes *n* and *m*, and the multiple DRFES g, an orthogonal time-slot is allocated to each DRFES g to transmit energy to each sensor node *n* and m to transmit its sensed water parameter information to the base station $g_{1}$. The sensor nodes *n* and *m* harness energy from the DRFES g in the downlink (DL) channels, while they transfer their independent acquired water parameter information to the base station $g_{1}$ in the uplink (UL) channels. In the TDMA model proposed for the MNMSMS WIPT system, since the sensor nodes are deployed close to the DRFES, an optimal shorter and equal EH time is allocated to the sensor nodes. Furthermore, since the sensor nodes are deployed in a predetermined manner, some of the sensor nodes are far from the base station $g_{1}$, resulting in a doubly near–far problem. This problem is addressed by allocating an optimal longer information-transmission time to the sensor nodes in order for them to have sufficient time to transmit their independent information.

### 5.2. Propagation Channel Model

In this paper, energy and power are used interchangeably. Since the WSN application environment is assumed to be static, the channel from a DRFES g to sensor nodes *n* and *m* is modelled as a quasi-static block fading model, and the channel gains are obtained as a feedback from the sensor nodes. The channels from sensor nodes *n* and *m* to the base station $g_{1}$ in the UL and the reversed DL channel from a DRFES g in the DL are represented with complex variables ${\tilde{a}}_{g,n}$ and ${\tilde{b}}_{g,n}$, and ${\tilde{f}}_{g,m}$ and ${\tilde{h}}_{g,n}$ for Network 1 and Network 2, respectively, while the channel power gains are $a_{g,n}={\left|{\tilde{a}}_{g,n}\right|}^{2}$ and $b_{g,n}={\left|{\tilde{b}}_{g,n}\right|}^{2}$, and $f_{g,m}={\left|{\tilde{f}}_{g,m}\right|}^{2}$ and $h_{g,m}={\left|{\tilde{h}}_{g,m}\right|}^{2}$ for Network 1 and Network 2, respectively. Also, the CSI is assumed to be known to the DRFES g, thus, energy is adaptively transferred based on the channel conditions. The DRFES g uses the CSI to access sensor node *n* and *m*, and transmit an optimal energy to each sensor node in an allocated time-slot.

The entire time-slots $q_{g}$ is dedicated to Network 1 for EH, while the entire time-slots $r_{g}$ is dedicated to Network 2 for EH. Network 1 transmits its information over the time-slots $\beta_{n}$, while Network 2 transmits its information over the time-slots $\Psi_{m}$. The operations of the two distinct networks based on the proposed TDMA protocol are described as follows.

Network 1: In each given EH time-slot $q_{g}$, according to the TDMA model presented in Figure 2, the first time-slot with a length of $0\leq q_{1}\leq 1$ is assigned to a DRFES g to transfer energy to sensor node *n* in the DL, while the time allocated to sensor node *n* to transmit its information in the UL to the base station $g_{1}$, over channel $a_{1,n}$, is represented with $\beta_{n}$, *n* = 1, *…*, *N*, with a length of $0\leq \beta_{n}\leq 1$. Therefore, the time allocated to a DRFES g to transmit energy to sensor node *n* in Network 1 in the DL and the allocated time to sensor nodes *n* to transmit their independent information in the UL is defined by (1) as:

(1) $$\overset{G}{\underset{g=1}{\sum }}q_{g}+\overset{N}{\underset{n=1}{\sum }}\beta_{n}\leq 1$$

The received power at sensor node *n* from a DRFES is given by (2) as:

(2) $$x_{g,n}=\sqrt{b_{g,n\;}}x_{A,g}+z_{n},\;n=1,\ldots ,N$$

where $x_{g,n}$ and $z_{n}$ represents the received signal and the background noise at sensor node *n* from a DRFES g respectively. $x_{A,g}$ denotes an arbitrary complex random signal DRFES g transmit which satisfies $E\left[{\left|x_{A,g\;}\right|}^{2}\right]=P_{A,g}$, where $P_{A,g}$ represents the transmit power of a DRFES g. The notation E[.] denotes an expectation operator, while [.] represents the magnitude of an argument. The transmit power $P_{A,g}$ is assumed to be large enough, thus, the background noise at the receiver *n* is negligible. Therefore, the amount of energy sensor node *n* harvest in the DL in each time-slot from a DRFES g is expressed by (3) as:

(3) $$E_{g,n}=\eta_{n}P_{A,g\;}b_{g,n}q_{g},\;g=1,\ldots ,G,\;n=1,\ldots ,N$$

Furthermore, the total energy sensor node *n* harvest from the DRFES g is given by (4) as:

(4) $$E_{n}=\eta_{n}\overset{G}{\underset{g=1}{\sum }}\;P_{A,g\;}b_{g,n}q_{g},\;n=1,\ldots ,N$$

where $\eta_{n}$ is the energy-harvesting efficiency of sensor node *n*, $0\leq \eta_{n}\leq 1$, *n* = 1, 2, *…*, *N.* It is assumed for convenience that $\eta_{1}=\ldots =\eta_{N}=\eta$ . After harvesting energy in the DL phase, a fixed fraction of the energy harvested based on (4) is used by each sensor node *n* to transmit its independent information in the UL to the base station $g_{1}$. The average transmit power of sensor node *n* for information transmission, is denoted by $P_{n}$ and is calculated using (5):

(5) $$P_{n}=\frac{{ϛ}_{n}E_{n}}{\beta_{n}},\;n=1,\ldots ,N$$

where ${ϛ}_{n}$ denotes a fixed fraction of the energy harvested used by sensor node *n* to transmit its independent information in the UL to the base station. For the purpose of simplicity, it is assumed that, ${ϛ}_{n}=\ldots ={ϛ}_{N}=ϛ$, while the remaining portion of 1−ϛ is used for operating the sensor node *n* and its modules.

The received signal at the base station $g_{1}$ from sensor node *n* in each UL time-slot is expressed by (6) as:

(6) $$x_{g_{1},n}=\sqrt{a_{1,n\;}}x_{n}+z_{g_{1}},\;n=1,\ldots ,N$$

where $x_{g_{1},n}$ and $z_{g_{1}}$ are the received signal and the background noise at the base station $g_{1}$, respectively, $x_{n}$ denotes the random signal sensor node *n* transmit which satisfies E $\left[{\left|\;x_{n}\right|}^{2}\right]=P_{n}$. The channel capacity of the UL information transfer from sensor node *n* and the base station $g_{1}$ is given by (7) according to the Shannon’s law of channel capacity as [36]:

(7) $$C_{n}=\beta_{n}log_{2}\left(1+\frac{P_{n}a_{1,n}}{ᴦ\sigma^{2}}\right)$$

where $\beta_{n}$ denotes the information-transmission time-slot (channel bandwidth) for sensor node *n*, $P_{n}$ is the average transmit power of sensor node *n*, ᴦ denotes the signal-to-noise ratio (SNR) gap, $\sigma^{2}$ represents the thermal power noise due to the additive white Gaussian noise (AWGN) from the Shannon’s channel capacity. For sensor node *n* information transmission, the maximum achievable throughput in bits/s/Hz (bps/Hz) of sensor node *n* denoted by $R_{n}$ cannot exceed the channel capacity $C_{n}$ between the sensor node *n* and the base station $g_{1}$ using (8) and (9), therefore,

(8) $$R_{n}\leq \beta_{n}log_{2}\left(1+\frac{P_{n}a_{1,n}}{ᴦ\sigma^{2}}\right)$$

(9) $$R_{n\;}\left(q,\beta \right)=\beta_{n}log_{2}\left(1+\frac{P_{n}a_{1,n}}{ᴦ\sigma^{2}}\right)$$

(10) is derived by substituting (4) and (5) into (9) as:

(10) $$R_{n\;}\left(q,\beta \right)=\beta_{n}log_{2}\left(1+\alpha_{n}\frac{\sum_{g=1}^{G}q_{g}}{\beta_{n}}\right),\;n=1,\ldots ,N$$

where $q=\left[q_{1},\;q_{2},\;q_{3},\ldots ,q_{G}\right]$, $\beta =\left[\beta_{1},\ldots ,\beta_{n}\right]$, $\alpha_{n}$ denotes the received SNR at the base station $g_{1}$ as a result of the information transmitted by sensor node *n* and is expressed by (11) as:

(11) $$\alpha_{n}=\frac{{ϛ}_{n}\eta_{n}a_{1,n}\sum_{g=1}^{G}\;P_{A,g\;}b_{g,n}q_{g}}{ᴦ\sigma^{2}},\;n=1,\ldots ,N$$

Therefore, the sum-throughput of all sensor node *n* is given by (12) as:

(12) $$R_{sum\;}\left(q,\beta \right)=\overset{N}{\underset{n=1}{\sum }}R_{n\;}\left(q,\beta \right),\;n=1,\ldots ,N$$

Network 2: In each given EH time-slot $r_{g}$, according to the TDMA model presented in Figure 2, the first time-slot with a length of $0\leq r_{1}\leq 1$ is assigned to a DRFES g to transfer energy to sensor node *m* in the DL, while the time allocated to sensor node *m* to transmit its information in the UL to the base station $g_{1}$, over channel $f_{1,m}$, is represented with $\Psi_{m}$, *m* = 1, 2, *…*, *M*, with a length of $0\leq \Psi_{m}\leq 1$. Therefore, the time allocated to a DRFES g to transmit energy to sensor node *m* in Network 2 in the DL and the time allocated to all the sensor nodes *n* to transmit their independent information in the UL is given by (13) as:

(13) $$\overset{G}{\underset{g=1}{\sum }}r_{g}+\overset{M}{\underset{m=1}{\sum }}\Psi_{m}\leq 1$$

The received power at sensor node *m* from a DRFES is given by (14) as:

(14) $$x_{g,m}=\sqrt{h_{g,m\;}}x_{A,g}+z_{m},\;m=1,\ldots ,M$$

The amount of energy sensor node *m* harvest in the DL in each time-slot from a DRFES g is expressed by (15) as:

(15) $$E_{g,m}=\eta_{m}P_{A,g\;}h_{g,m}r_{g},\;g=1,\ldots ,G,\;m=1,\ldots ,M$$

Therefore, the total energy sensor node *m* harvest from the DRFES g is given by (16) as:

(16) $$E_{m}=\eta_{m}\overset{G}{\underset{g=1}{\sum }}\;P_{A,g\;}h_{g,m}r_{g},\;m=1,\ldots ,M$$

where $\eta_{m}$ is the energy-harvesting efficiency of sensor node *m*, $0\leq \eta_{m}\leq 1$, *m* = 1, 2, *…*, *M.* It is assumed for convenience that $\eta_{1}=\ldots =\eta_{M}=\eta$. After harvesting energy in the DL phase, a fixed fraction of the energy harvested based on (16) is used by each sensor node *m* to transmit its independent information in the UL to the base station $g_{1}$. The average transmit power of sensor node *m*, $P_{m}$, is calculated using (17):

(17) $$P_{m}=\frac{{ϛ}_{m}E_{m}}{\Psi_{m}},\;m=1,\ldots ,M$$

where ${ϛ}_{m}$ denotes a fixed fraction of the energy harvested used by sensor node *m* to transmit its independent information in the UL to the base station. For the purpose of simplicity, it is assumed that ${ϛ}_{m}=\ldots ={ϛ}_{M}=ϛ$, while the remaining portion of 1−ϛ is used for operating the sensor node *n* and its modules.

From (16) and (17), the channel capacity of the UL information transmission from sensor node *m* and the base station $g_{1}$ is given by (18) according to the Shannon’s law of channel capacity as:

(18) $$R_{m\;}\left(r,\Psi \right)=\Psi_{m}log_{2}\left(1+\gamma_{m}\frac{\sum_{g=1}^{G}r_{g}}{\Psi_{m}}\right),\;m=1,\ldots ,M$$

where $r=\left[r_{1},\;r_{2},\;r_{3},\ldots ,r_{G}\right]$, $\Psi =\left[\Psi_{1},\ldots ,\Psi_{m}\right]$, $\Psi_{m}$ denotes the information-transmission time-slot (channel bandwidth) for sensor node *m*, $\gamma_{m}$ denotes the received SNR at the base station $g_{1}$ as a result of the information transmitted by sensor node *m* and is expressed by (19) as:

(19) $$\gamma_{m}=\frac{{ϛ}_{m}\eta_{m}f_{1,m}\sum_{g=1}^{G}\;P_{A,g\;}h_{g,m}r_{g}}{ᴦ\sigma^{2}},\;m=1,\ldots ,M$$

Therefore, the sum-throughput of sensor node *m* is given by (20) as:

(20) $$R_{sum\;}\left(r,\Psi \right)=\overset{M}{\underset{m=1}{\sum }}R_{m\;}\left(r,\Psi \right),\;m=1,\ldots ,M$$

## 6. Sum-Throughput Maximization Problem

To ensure a fair allocation of information-transmission rates between sensor nodes *n* and *m*, and to also reduce energy-harvesting unfairness among sensor nodes *n* and *m*, a sum-throughput maximization technique is employed to jointly optimize the energy-harvesting time and the information-transmission rates allocation of sensor nodes *n* and *m*. By so doing, the energy-harvesting efficiency and the information-transmission rates allocation among sensor nodes *n* and *m* are improved. The formulation of the throughput maximization problem is derived from (1) and expressed by (21) as:

(P1):

(21) $$\underset{q,\beta ,r,\Psi }{\max}R_{sum\;}\left(q,\beta \right)+R_{sum\;}\left(r,\Psi \right)$$

subject to:

(22) $$\overset{G}{\underset{g=1}{\sum }}q_{g}+\overset{G}{\underset{g=1}{\sum }}r_{g}+\overset{N}{\underset{n=1}{\sum }}\beta_{n}+\overset{M}{\underset{m=1}{\sum }}\Psi_{m}\leq 1$$

(23) $$q_{g}\geq 0,\;g=1,\ldots ,G$$

(24) $$r_{g}\geq 0,\;g=1,\ldots ,G$$

(25) $$\beta_{n}\geq 0,\;n=1,\ldots ,N$$

(26) $$\Psi_{m}\geq 0,\;m=1,\;\ldots ,M$$

where (21) is the objective function, constraints (22) represents the energy harvesting and information-transmission time scheduling, (23)–(26) represent the non-negative constraints for the decision variables. The unknown variables in (P1) are *q*, *β*, *r*, *Ψ*. The problem in (21) is a non-convex problem due to the *log* function in (10) and (18). The transformation of the problem to a convex problem was achieved by employing the Lagrangian dual-decomposition method due to its lower complexity. The transformation is described in Appendix A. The transformed problem (P1′) can be using any convex optimization method. Furthermore, to address energy-harvesting unfairness among the sensor nodes due to the problem transformation, a new problem (P2) is formulated to ensure optimal values of q and r denoted as q* and r*. The optimal values of q* and r* are used in (P1) to ensure energy-harvesting fairness among sensor nodes *n* and *m*. The energy-harvesting unfairness minimization problem is formulated in (27) as follows:

(P2):

(27) $$\underset{q^{*},r^{*}}{\min}E\left[{\left(E_{n}-{\bar{E}}_{n}\right)}^{2}+{\left(E_{m}-{\bar{E}}_{m}\right)}^{2}\right]$$

subject to:

(28) $$\sum_{g=1}^{G}q_{g}+\sum_{g=1}^{G}r_{g}=1$$

(29) $$q_{g}\geq 0,\;g=1,\ldots ,G$$

(30) $$r_{g}\geq 0,\;g=1,\ldots ,G$$

where ${\bar{E}}_{n}$ and ${\bar{E}}_{m}$ denotes the minimum energy harvested by sensor nodes *n* and *m*. ${\bar{E}}_{n}$ is calculated using (31):

(31) $$E_{n}=E\left(E_{n}\right)=\frac{\sum_{n=1}^{N}E_{n}}{N}$$

${\bar{E}}_{m}$ is calculated using (32):

(32) $$E_{m}=E\left(E_{m}\right)=\frac{\sum_{m=1}^{M}E_{m}}{M}$$

(P2) depends on variables *q*, and *r*, these variables are yet unknown. To solve for subsequent set of $E_{n}$, *n* = 1, *…*, *N* and $E_{m}$, *m* = 1, *…*, *M*, non-zero arbitrary values can be assumed for $q_{g}$ and $r_{g}$. Finding q* and r* is achieved as described in the proof for the energy-harvesting unfairness minimization problem, given in Appendix B.

Furthermore, a multiple DRFES selection algorithm is proposed and used with the sum-throughput maximization method for the multi-network, multi-sensor WIPT system for EH efficiency and fair information-transmission rate allocations. In Section 5, through simulation experiments, the algorithm is shown to be computationally efficient. Moreover, to investigate the information-transmission rate fairness between sensor nodes *n* and *m*, the equation of Jain’s fairness index is employed and given by (33). According to Jain’s fairness equation [37]:

(33) $$J=\frac{{\left(\sum_{i=1}^{u}R_{u\;}\left(\delta \right)\right)}^{2}}{u.\sum_{i=1}^{u}{\left(R_{u\;}\left(\delta \right)\right)}^{2}}$$

where u=n+m denotes the summations of sensor nodes *n* and *m*, δ=(q+β)+(r+Ψ) denotes the time-length for Network 1, that is, sensor nodes *n*, and Network 2, that is, sensor nodes *m*, $R_{u}\left(\delta \right)=R_{n}\left(q,\beta \right)+R_{m}\left(r,\Psi \right)$ denotes the summation of the sum-throughput of Network 1 and Network 2. Also, the worst case and the best case for sensor nodes *n* and *m* is defined by $\frac{1}{U}\leq J_{FI}\leq 1$, $\frac{1}{U}$ denotes a minimum fairness rate, while 1 denotes a maximum fairness rate.

## 7. Energy Harvesting and Information-Transmission Rate Joint Optimization

Since multiple DRFES and multiple sensor nodes are considered in the proposed WIPT system, to address the problem of unfairness in EH among the sensor nodes and also to improve the information-transmission rates of the sensor nodes, the energy harvesting and the information-transmission timings of the MNMSMS WIPT system are jointly optimized so as to increase fairness in the energy harvested from the DRFES among the sensor nodes. To achieve this, the proposed Algorithms 1 and 2 are employed to provide optimal energy-harvesting timings and efficient information-transmission rates, thus, maximizing the achievable throughput of the WIPT system. Since the DRFES are connected to a controller, Algorithm 1 is implemented at the controller for optimal selection and allocation of the DRFES to the sensor nodes in an efficient manner.

**Algorithm 1.** Selection of DRFES and efficient allocation.Require: $\left\{n_{1},\;n_{2},\ldots ,\;N\right\}$
⊳
sensor nodes n; $\left\{m_{1},\;m_{2},\ldots ,\;M\right\}$
⊳
sensor nodes m;   $\left\{g_{1},\;g_{2},\ldots ,\;G\right\}$
⊳
DRFESs gEnsure: $q_{g}^{*}$, $r_{g}^{*}$, g=1, 2, …,G
⊳
optimal
energy harvesting time1:   for g=1:G do2:   set g to ON3:   for n=1:N do4:    if g is the nearest to n5:    set g to ON and n harvest energy from g using Eqation (4)6:    else7:      continue8:    end if9:   end for10:   set g to OFF11: end for12: for g=1:G do13: set g to ON14: for m=1:M do15: if g is the nearest to m,16:   set g to ON and m harvest energy from g using Eqation (16)17: else18:   continue19: end if20: end for21: set g to OFF22: *end for**end*

In the multi-network WIPT system, Algorithm 1 is used to optimally select and switch on and off the DRFES. It also considers the number of sensor nodes *n* and *m*, and the available DRFES g in the system in each time-slot. Algorithm 1 is responsible for optimally allocating a DRFES g to sensor nodes *n* and *m* by firstly considering the nearest DRFES g to the sensor nodes, this is important so as to increase fairness in the energy harvested by the sensor nodes from the dedicated RF energy-harvesting sources g in each energy-harvesting phase. Based on the nearness of the DRFES to sensor nodes *n* and *m*, Algorithm 1 switches a DRFES g on in an allocated EH time-slot. Furthermore, Algorithm 2 is employed to ensure a fair information-transmission rate allocation among sensor nodes *n* and *m*.

**Algorithm 2.** Efficient information transmission in the uplink.Require: $\left\{n_{1},\;n_{2},\ldots ,\;N\right\}$
⊳
sensor nodes n; $\left\{m_{1},\;m_{2},\ldots ,\;M\right\}$
⊳
sensor nodes m;  $\left\{g_{1},\;g_{2},\ldots ,\;G\right\}$
⊳
DRFESs g gn ⊳    energized sensor node in n    gm ⊳
energized sensor node in m1: for each gn
*in*
n do2: *transfer information in the UL to*
$g_{1,}$
using
*Equation (5) over*
$a_{1,n}=10^{-3}d_{g,n}^{-\rho },\;g=1,\;2,\;\ldots ,G\;n=1,\;2,\;\ldots ,N$3: end for4: for each gm
*in*
m do5: *transfer information in the UL to*
$g_{1,}$
using
*Equation (17) over*
$f_{1,m}=10^{-3}d_{g,m}^{-\rho },\;g=1,\;2,\;\ldots ,G,\;m=1,\;2,\;\ldots ,M$6: end for*end*

## 8. Simulation and Discussion

The multi-network WIPT system was configured using the simulation parameters in Table 1. The configuration of the multi-network WIPT system includes sensor nodes *n* and *m*, contained in Network 1 and Network 2, respectively. The DRFES g in the WIPT system are deployed in a predetermined manner in an illustrative environment of 21 m by 21 m. One of the DRFES is randomly selected as the base station for the UL, assumed to be at a fixed distance, D_UL_, from both the networks. Since there is a channel reciprocity in Network 1 and Network 2 in the DLs and in the ULs, we assume the channel model of $a_{g,n}=b_{g,n}=10^{-3}d_{g,n}^{-\rho },\;n=1,\;2,\;\ldots ,N$ for Network 1, and $f_{g,m}=h_{g,m}=10^{-3}d_{g,m}^{-\rho },$ m = 1, 2, …, M for Network 2, where $d_{g,n}$ stands for the distance between sensor node *n* and the DRFES g in meters, $d_{g,m\;}$ stands for the distance between sensor *m* and the DRFES g in meters, and ρ denotes the path loss exponent.

The same simulation parameters have been assumed in the SNMSMS work described in [22], which will be used as a state-of-the-art baseline for comparing the results obtained in the proposed MNMSMS of this work.

**Figure 3:**
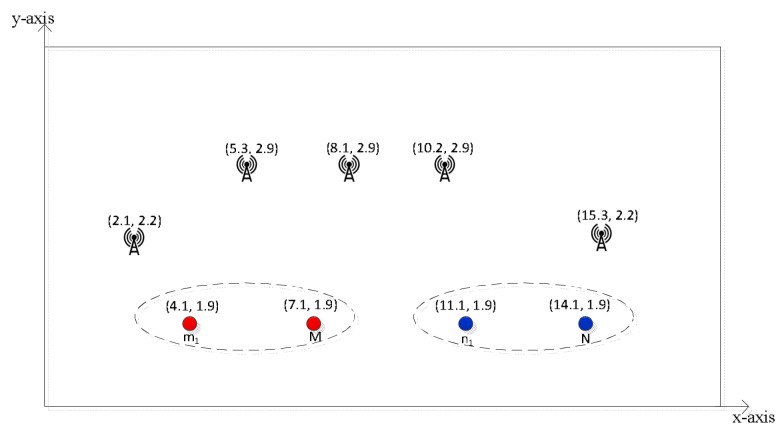
Example of a network structure.

An example MNMSMS simulation with five DRFES could, for instance, produce coordinates of (8.1 m, 2.9 m) for $g_{1}$, (2.1 m, 2.2 m) for $g_{2},$ (5.3 m, 2.9 m) for $g_{3}$, (10.2 m, 2.9 m) for $g_{4}$, and (15.3 m, 2.2 m) for $g_{5}$. For the purpose of showing the coordinates of the DRFES, an example of a network structure is given in Figure 3.

### 8.1. Results and Discussion

The proposed algorithms presented in Section 5 were implemented for simulation experiments on the proposed MNMSMS system for various configuration instances, while evaluating the achievable average sum-throughput and the fairness of the resulting system. In each run, the system considered the number of sensor nodes and the number of the DRFES as input parameters. For each simulation experiment, the total number of sensor nodes *n* and *m* was denoted by *k*. Also, for each run, different placements of the networks *n* and *m* were efficiently generated by considering the nearest DRFES to the sensor nodes for optimal allocation. In order to compare the proposed WIPT system with the existing state-of-the-art system in [22], a total number of sensor nodes *k* equal to two and three were considered for different numbers of DRFES, ranging between one and five.

#### 8.1.1. Effect of Run Time on the System-Achievable Sum-Throughput and Fairness

**Figure 4:**
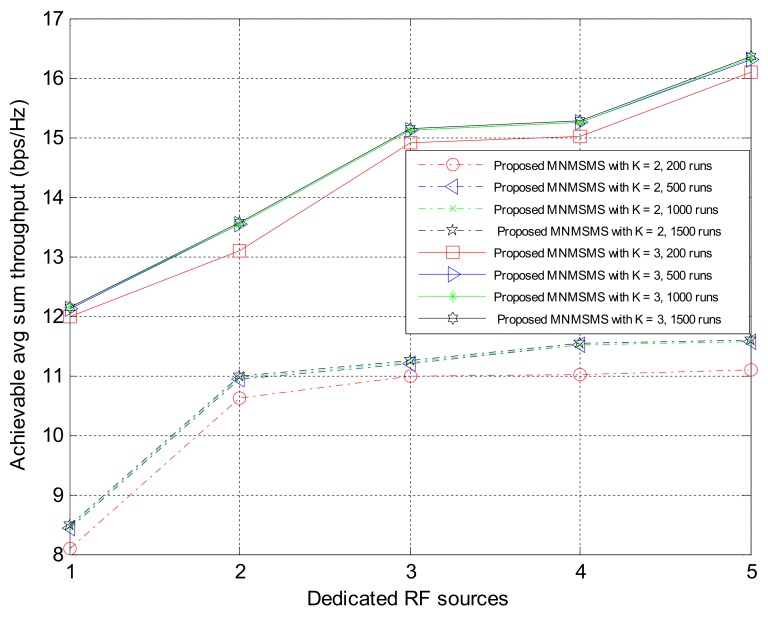
The impact of run time on the average system-achievable sum-throughput.

Ensuring convergence of the optimization algorithms for efficiently finding and allocating the best DRFES for energy harvesting is important, and therefore the effect of the runtime on the system outputs are considered. Figure 4 shows the average achievable sum-throughput rate, of the system depending on the total number of simulation runs. Both Algorithms 1 and 2 were activated for all simulation runs. For these runs a two-sensor system and a three-sensor system configuration are used, while the number of DRFES were varied, to a maximum of five. It was established from Figure 4 that a larger number of simulation runs improved the system throughput rate of a two-sensor system with a maximum difference of only 0.3%, and a three-sensor system with a maximum difference of 0.3%. This is an indication that the two-sensor system and the three-sensor system have the same rate of convergence, due to the number of the sensor nodes in the optimization problem. From these results, it can be deduced that averaging the system outputs over 500 runs provide reasonable results.

#### 8.1.2. Effect of Network Distance from the Base Station on the System-Achievable Sum-Throughput

**Figure 5:**
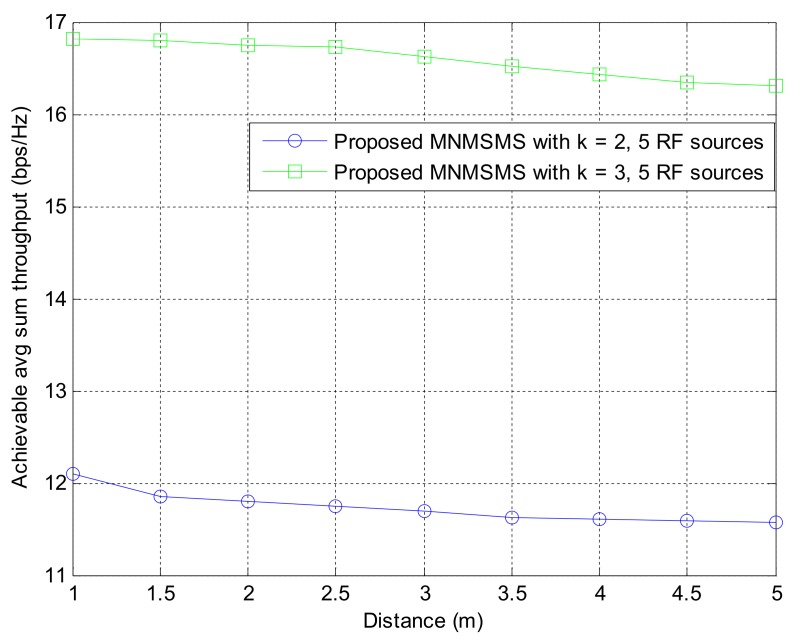
The impact of different networks distance from the base station on the system-achievable throughput rate.

This section studies the effect of the distance of networks to the base station on the achievable sum-throughput rate in a two-sensor system and a three-sensor system powered by five power sources over an average of 500 run times. The achievable average sum-throughput of the system slightly deceases as the distance of the networks from the base station increases, as shown in Figure 5. This establishes that the rate at which the system throughput decreases with distance is small.

#### 8.1.3. Comparison of Sum-Throughput and Fairness Performance for Different System Configurations

**Figure 6:**
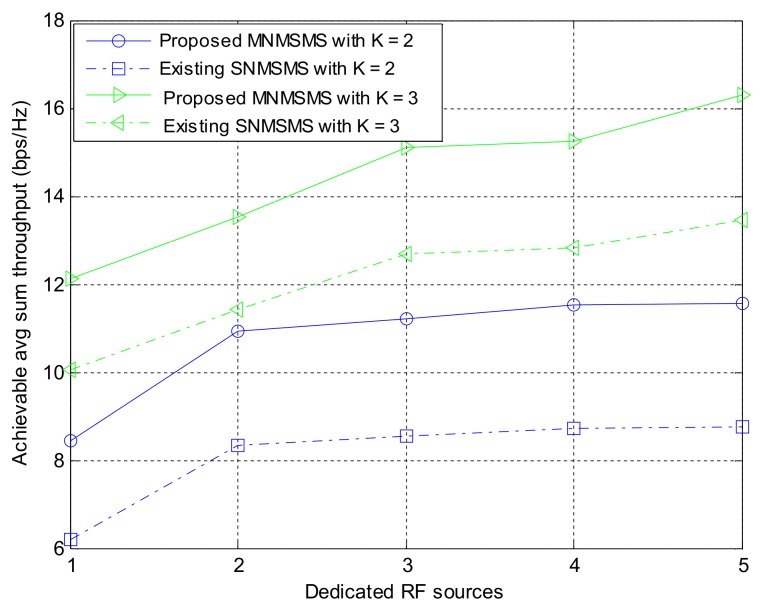
Achievable average sum-throughput against number of dedicated RF sources.

**Figure 7:**
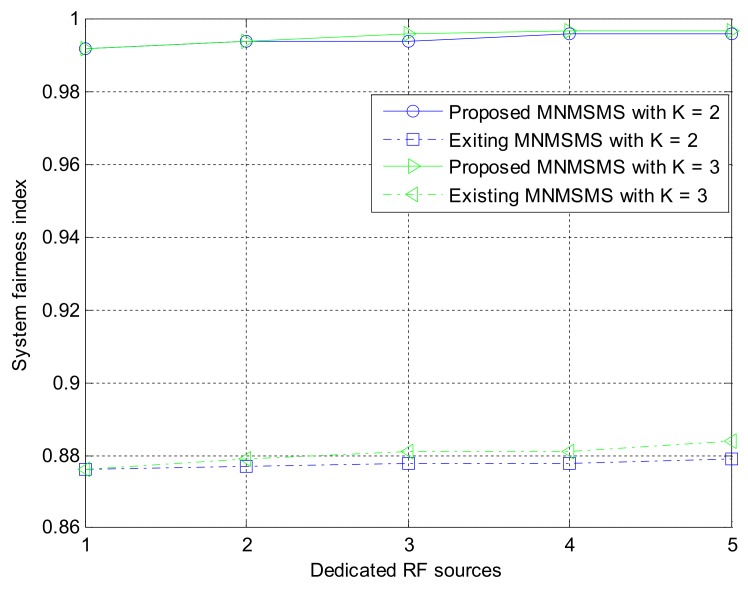
System fairness index against number of dedicated RF sources.

Simulation experiments were performed on the proposed MNMSMS system and the existing SNMSMS system for various configuration instances, where the results included the achievable average sum-throughput and the fairness of the systems. In each run, the system considered the number of sensor nodes and the number of the DRFES as input parameters. For each simulation experiment, the total number of sensor nodes *n* and *m* was denoted by *k*. Also, for each run, different placements of the networks *n* and *m* were efficiently generated by considering the nearest DRFES to the sensor nodes for optimal allocation. Both Network 1 and Network 2 are considered at a distance of 5 m to the base station. Each simulation experiment was averaged over a run of 500 instances in time. Figure 6 describes the achievable average sum-throughputs of the proposed multi-network WIPT system and the single-network WIPT system, while Figure 7 presents the fairness of the multi-network and the single-network systems. The MNMSMS system produced a better achievable sum-throughput and fairness value than the existing SNMSMS system. The improvements are due to the efficient selection and allocation of the DRFES using Algorithms 1 and 2. For example, when *k* is set to two with one DRFES, the MNMSMS system produced an average sum-throughput of 8.44 bps/Hz and a fairness index of 0.992, while the data published in [22] for the SNMSMS system are an average sum-throughput of 6.18 bps/Hz and a fairness index of 0.876. These results represent significant improvements of 36% in the system throughput rate, and 13% in the fairness index. Also, when *k* is set to three with a DRFES of one, the MNMSMS system produced an average sum-throughput of 12.11 bps/Hz and a fairness index of 0.992, while the published data for the SNMSMS system are an average sum-throughput of 10.07 bps/Hz and a fairness index of 0.876 [22]. These results represent significant improvements of 20% in the system throughput rate, and 13% in the fairness index.

Also, the results conform with the mathematical analysis in (4), (5), (10), (16)–(18), because the system throughput is influenced by the amount of energy harvested by the sensor nodes. As shown in Figure 7, the problem of the doubly near–far condition between sensor nodes in the existing WIPT systems was efficiently handled by Algorithms 1 and 2 through the increase in the influx of dedicated RF power sources to the proposed system. Furthermore, the fairness results presented in Figure 7 for the proposed system significantly improved compared to the fairness of the existing state-of-the-art system. This was as a result of the quality channel experienced in the proposed system, where the static wireless channels between the DRFES and the sensor nodes are always constant, and this has favoured the fair allocation of EH time and information-transmission rate to each sensor node in the network. Consequently, the overall system throughput rate and fairness rate are improved.

#### 8.1.4. Comparison of the System Performance Dependent on Network Distance from the Base Station

**Figure 8:**
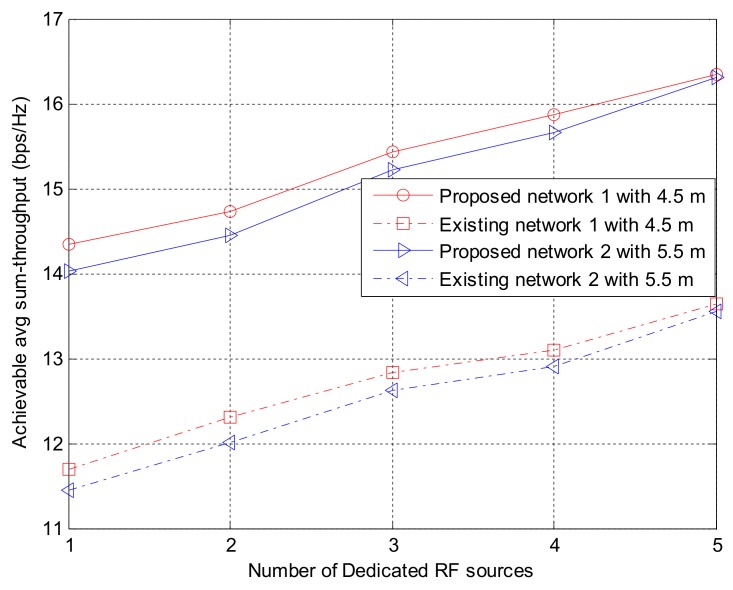
The impact of different network distance D_UL_ on the system-achievable throughput rate.

This section investigated the effect of networks distance in the uplink, D_UL_. To achieve this, an equal distance among two groups of network in the MNMSMS was considered. One network simulation was considered at a distance of 4.5 m to the base station, while another network simulation was considered at a distance of 5.5 m to the base station in a WIPT system with three sensor nodes, and powered by five 3W DRFES. As shown in Figure 8, the difference in the network distance slightly influenced the system-achievable throughput rate. This decrease is due to the system wireless channel conditions. Consequently, the distance of a network to the base station in a MNMSMS WIPT system contributes to the achievable throughput rate of the system.

**Figure 9:**
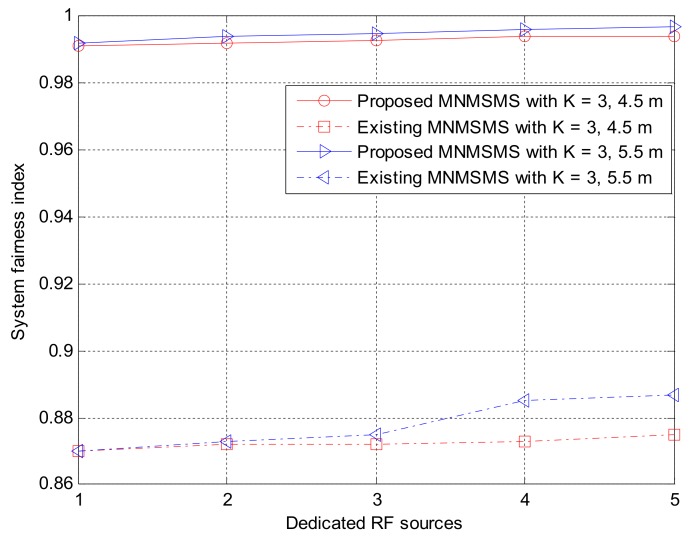
The impact of different network distance D_UL_ on the system fairness index.

The results obtained were also compared with the existing state-of-the-art WIPT system to further emphasize the contributions of the proposed algorithms. The average difference between the throughput rates as a function of D_UL_ of the MNMSMS and SNMSMS are 1.4% and 1.6% for distances 4.5 m and 5.5 m. This is an indication that the MNMSMS system has a better rate of convergence compared to the SNMSMS system, due to the efficiency of Algorithms 1 and 2 in handling fairness in EH and information-transmission rate allocation. Figure 9 presents the system fairness index. Once again, average fairness difference with distance of the MNMSMS and SNMSMS are 0.2% and 0.6%. This is due to the efficiency of Algorithms 1 and 2 in ensuring fairness among the sensor nodes in the MNMSMS system

#### 8.1.5. Effect of Transmission Power on the System-Achievable Sum-Throughput

**Figure 10:**
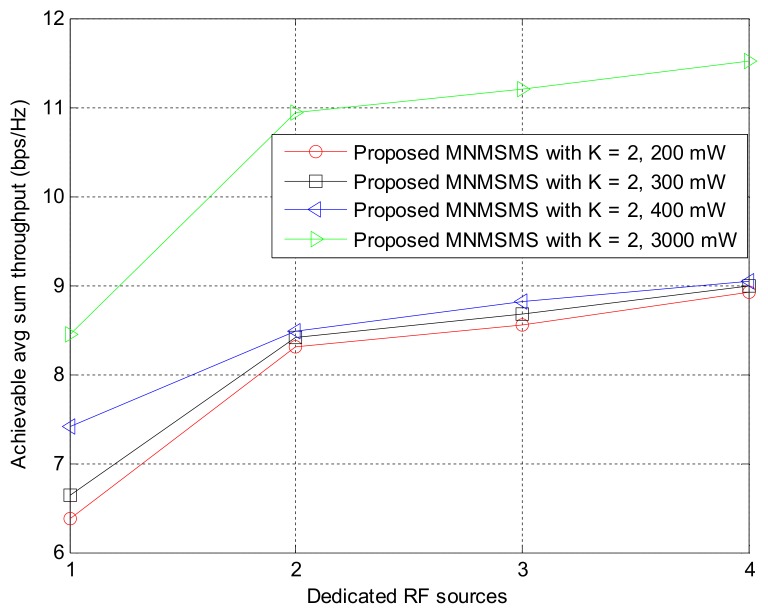
The impact of transmit power on the system-achievable throughput rate.

The effect of different transmit power on the system’s achievable sum-throughput rate is investigated in Figure 10. The system is configured with *k* equal to 2 and powered by four DRFES. The transmit power of the DRFES is set to 200 mW, 300 mW, 400 mW, and 3000 mW. Based on the different values of transmit power, a significant increase in the system’s throughput rate was observed as the transmit power of the DRFES was increased from 200 mW, 300 mW, 400 mW to 3000 mW. This is an indication that the transmit power of the DRFES directly influences the performance of the system. From the result, it can be observed that even for transmit power as low as 200 mW, the achieved throughput rate result is adequate, which means that the proposed system is energy-efficient for sustainable network communications.

## 9. Conclusions

This work has provided a solution to the problem of unfairness in energy harvesting and unequal information-transmission rate allocation, also known as the doubly near–far problem, in a multi-network, multi-sensor and multi-source WIPT system in a static environment by studying efficient resource allocation for increased system performance. To achieve this, new efficient optimization algorithms were proposed. The proposed algorithms have revealed improved and interesting throughput rate and fairness results compared to the existing state-of-the-art algorithm. The newly proposed system provided a better fairness index among the sensor nodes in the system, and a great transmission throughput rate. The newly proposed algorithms had improvements of 36% in the system throughput rate, and a fairness index of 13% for a two-sensor system. These results represent significant improvements in the system throughput rate and fairness index. Also, the proposed WIPT system is energy-efficient, and ensures sustainable communication.

## Figures and Tables

**Table 1:** Assumed simulation parameters.

Parameter	Value
Transmission power of the dedicated RF sources	3000 mW
Noise power	−114 dBm
Energy-harvesting efficiency	0.5
Fraction of energy used for information transmission	0.5
Signal-to-noise gap	1.5
Radio frequency (RF) transceiver frequency	915 MHz
Bandwidth	1 MHz
Path loss exponent	2
Medium access control	IEEE 802.15.4

## Appendix A

Proof of Convexity for Problem P1 Transformation.

The optimization problem (P1) in (21) is a non-convex problem because $R_{sum}$ contains a *log* function. The transformation of (P1) to a convex problem was achieved through the application of the Lagrangian dual decomposition method to obtain a new problem P1′. The Lagrangian of (21) is given as follows:

From (10) and (18),

(A1) $$R_{n\;}\left(q,\beta \right)=\frac{\beta_{n}}{\mathrm{In}2}\mathrm{In}\left(1+\alpha_{n}\frac{\sum_{g=1}^{G}q_{g}}{\beta_{n}}\right),\;n=1,\ldots ,N$$

(A2) $$R_{m\;}\left(r,\Psi \right)=\frac{\Psi_{m}}{\mathrm{In}2}\mathrm{In}\left(1+\gamma_{m}\frac{\sum_{g=1}^{G}r_{g}}{\Psi_{m}}\right),\;m=1,\ldots ,M$$

where:

$$q=q_{1},\;q_{2},\;\ldots ,q_{G},\;\beta =\beta_{1},\beta_{2},\ldots ,\beta_{N}$$

$$r=r_{1},\;r_{2},\;\ldots ,r_{G},\;\Psi =\Psi_{1},\Psi_{2},\ldots ,\Psi_{M}$$

The sum-throughput of all sensor nodes *n* and *m*, that is, $R_{n,m}$ or $R_{sum}$ is:

$$R_{n\;,m}\;\left(q,\beta ,r,\;\psi \right)=\sum_{n=1}^{N}R_{n}\;\left(q,\beta \right)+\sum_{m=1}^{M}R_{m}\;\left(r,\psi \right)$$

To show that (P1) is concave, let the Lagrangian multiplier be λ, where λ ≥ 0 is associated to constraint (22). The Lagrangian of (P1) is given by (P1′).

(P1′)

$$L\left(q,r,\beta ,\Psi ,\lambda \right)=R_{n,m\;}\left(q,r,\beta ,\Psi \right)+\lambda \left(\overset{G}{\underset{g=1}{\sum }}q_{g}+\overset{G}{\underset{g=1}{\sum }}r_{g}+\overset{N}{\underset{n=1}{\sum }}\beta_{n}+\overset{M}{\underset{m=1}{\sum }}\Psi_{m}-1\right)$$

Substituting (A1) and (A2) into (P1′) gives:

$$L\left(q,r,\beta ,\Psi ,\lambda \right)=\frac{\beta_{n}}{\mathrm{In}2}\mathrm{In}\left(1+\alpha_{n}\frac{\sum_{g=1}^{G}q_{g}}{\beta_{n}}\right)+\frac{\Psi_{m}}{\mathrm{In}2}\mathrm{In}\left(1+\gamma_{m}\frac{\sum_{g=1}^{G}r_{g}}{\Psi_{m}}\right)$$

(A3) $$\lambda \left(\overset{G}{\underset{g=1}{\sum }}q_{g}+\overset{G}{\underset{g=1}{\sum }}r_{g}+\overset{N}{\underset{n=1}{\sum }}\beta_{n}+\overset{M}{\underset{m=1}{\sum }}\Psi_{m}-1\right)$$

By applying the Karush–Kuhn–Tucker (KKT) conditions to (A3), the following must hold:

$$\frac{\partial L}{\partial q_{g}}=0;\;\frac{\partial L}{\partial \beta_{n}}=0;\;\frac{\partial L}{\partial r_{g}}=0;\;\frac{\partial L}{\partial \Psi_{m}}=0;$$

Solving for $\frac{\partial L}{\partial q_{g}}=0$, find the derivative of *L* with respect to $q_{g}$,

(A4) $$L\left(q,r,\beta ,\Psi ,\lambda \right)=\frac{\beta_{n}}{\mathrm{In}2}\mathrm{In}\left(1+\alpha_{n}\frac{\sum_{g=1}^{G}q_{g}}{\beta_{n}}\right)+\lambda \left(\overset{G}{\underset{g=1}{\sum }}q_{g}\right)$$

(A5) $$\frac{\partial L}{\partial q_{g}}=\frac{\beta_{n}\alpha_{n}G}{\mathrm{In}2\left(\beta_{n}+\alpha_{n}\sum_{g=1}^{G}q_{g}\right)}+\lambda G$$

Solving for $\frac{\partial L}{\partial r_{g}}=0$, find the derivative of *L* with respect to $r_{g}$,

(A6) $$L\left(q,r,\beta ,\Psi ,\lambda \right)=\frac{\Psi_{m}}{\mathrm{In}2}\mathrm{In}\left(1+\gamma_{m}\frac{\sum_{g=1}^{G}r_{g}}{\Psi_{m}}\right)+\lambda \left(\overset{G}{\underset{g=1}{\sum }}r_{g}\right)$$

(A7) $$\frac{\partial L}{\partial r_{g}}=\frac{\Psi_{m}\gamma_{m}G}{\mathrm{In}2\left(\Psi_{m}+\gamma_{m}\sum_{g=1}^{G}r_{g}\right)}+\lambda G$$

Solving for $\frac{\partial L}{\partial \beta_{n}}$ = 0, find the derivative of *L* with respect to $\beta_{n}$,

(A8) $$L\left(q,r,\beta ,\Psi ,\lambda \right)=\frac{\beta_{n}}{\mathrm{In}2}\mathrm{In}\left(1+\alpha_{n}\frac{\sum_{g=1}^{G}q_{g}}{\beta_{n}}\right)+\lambda \left(\overset{N}{\underset{n=1}{\sum }}\beta_{n}\right)$$

(A9) $$\frac{\partial L}{\partial \beta_{n}}=\frac{1}{\mathrm{In}2}\mathrm{In}\left(1+\alpha_{n}\frac{\sum_{g=1}^{G}q_{g}}{\beta_{n}}\right)+\frac{1}{\mathrm{In}2}\frac{\beta_{n}\alpha_{n}G}{\beta_{n}+\alpha_{n}\sum_{g=1}^{G}q_{g}}$$

Solving for $\frac{\partial L}{\partial \Psi_{m}}=0$, find the derivative of *L* with respect to $\Psi_{m}$,

(A10) $$L\left(q,r,\beta ,\Psi ,\lambda \right)=\frac{\Psi_{m}}{\mathrm{In}2}\mathrm{In}\left(1+\gamma_{m}\frac{\sum_{g=1}^{G}r_{g}}{\Psi_{m}}\right)+\lambda \left(\overset{M}{\underset{m=1}{\sum }}\Psi_{m}\right)$$

(A11) $$\frac{\partial L}{\partial \Psi_{m}}=\frac{1}{\mathrm{In}2}\mathrm{In}\left(1+\gamma_{m}\frac{\sum_{g=1}^{G}r_{g}}{\Psi_{m}}\right)+\frac{1}{\mathrm{In}2}\;\frac{\Psi_{m}\gamma_{m}G}{\Psi_{m}+\gamma_{m}\sum_{g=1}^{G}q_{g}}$$

Solving for $\frac{\partial L}{\partial \lambda }$ = 0, find the derivative of *L* with respect to λ,

(A12) $$L\left(q,r,\beta ,\Psi ,\lambda \right)=\lambda \left(\overset{G}{\underset{g=1}{\sum }}q_{g}+\overset{G}{\underset{g=1}{\sum }}r_{g}+\overset{N}{\underset{n=1}{\sum }}\beta_{n}+\overset{M}{\underset{m=1}{\sum }}\Psi_{m}-1\right)$$

(A13) $$\frac{\partial L}{\partial \lambda }=\left(\overset{G}{\underset{g=1}{\sum }}q_{g}+\overset{G}{\underset{g=1}{\sum }}r_{g}+\;\overset{N}{\underset{n=1}{\sum }}\beta_{n}+\overset{M}{\underset{m=1}{\sum }}\Psi_{m}-1\right)$$

Thus, (P1′) is a concave optimization problem. Consequently, any convex optimization method can be employed to address (P1′).

## Appendix B

Proof of Energy-Harvesting Unfairness Minimization.

From constraint (28):

(A14) $$\overset{G}{\underset{g=1}{\sum }}q_{g}+\overset{G}{\underset{g=1}{\sum }}r_{g}=1$$

we have,

(A15) $$\sum_{g=1}^{G}\;P_{A,g\;}b_{n}q_{g}+\sum_{g=1}^{G}\;P_{A,g\;}h_{m}r_{g}$$

Putting (A15) in (4) and (16) gives:

(A16) $$E_{n,m}=\eta_{n}\sum_{g=1}^{G}\;P_{A,g\;}b_{n}q_{g}+\eta_{m}\sum_{g=1}^{G}\;P_{A,g\;}h_{m}r_{g},\;n=1,\;2,\;\ldots ,\;N,\;m=1,\;2,\;\ldots ,\;M$$

Let $E_{n,m}^{*}$ be the optimal values for $E_{n}$ and $E_{m}$ due to $q_{g}^{*}$ and $r_{g}^{*}$.

Since $q_{g}^{*}$ and $r_{g}^{*}$ represent the optimal values for $q_{g}$ and $r_{g}$. Therefore, from (A16), we have:

(A17) $${E^{*}}_{n,m}=\eta_{n}\sum_{g=1}^{G}\;P_{A,g\;}b_{n}q_{g}^{*}+\eta_{m}\sum_{g=1}^{G}\;P_{A,g\;}h_{m}r_{g}^{*},\;n=1,\;2,\;\ldots ,\;N,\;m=1,\;2,\;\ldots ,\;M$$

From (A17), $q_{g}^{*}$ and $r_{g}^{*}$ give optimal values for subsequent values of $E_{n,m}^{*}$.
